# Bone Hardness of Different Anatomical Regions of Human Radius and its Impact on the Pullout Strength of Screws

**DOI:** 10.1111/os.12436

**Published:** 2019-03-25

**Authors:** Wei‐wei Wu, Yan‐bin Zhu, Wei Chen, Sheng Li, Bing Yin, Jian‐zhao Wang, Xiao‐juan Zhang, Guo‐bin Liu, Zu‐sheng Hu, Ying‐ze Zhang

**Affiliations:** ^1^ Department of Orthopaedic Surgery The Third Hospital of Hebei Medical University Shijiazhuang China; ^2^ Key Laboratory of Biomechanics of Hebei Province Shijiazhuang China

**Keywords:** Bone hardness, Microindentation hardness test, Pullout strength, Radius

## Abstract

**Objective:**

To investigate the bone hardness of different anatomical regions of the human radius and its impact on the pullout strength of screws.

**Methods:**

Fresh radius bones were obtained from three donated cadavers. They were divided into three parts: proximal metaphysis, shaft, and distal metaphysis. The proximal metaphysis contains the head, neck, and radial tuberosity. The distal metaphysis includes the palmaris radius and the styloid process. The shaft of the radius was divided into nine segments of equal length. The bone hardness of three radiuses, one from each cadaver, was measured by Vickers microindentation hardness tests, and the screw pullout strength was examined in the other three radiuses using a materials testing machine. The trend between radius hardness and pullout strength was analyzed by using an analysis of variance randomized block design. Pearson correlation analysis was performed to evaluate the linear correlation between the bone hardness and the pullout strength of the human radius.

**Results:**

The mean hardness ranged from 33.30 HV (the head) to 43.82 HV (the diaphysis). The hardest part of the radius was the shaft, with a value of 42.54 ± 5.59 HV. The proximal metaphysis had a hardness value of 34.15 ± 6.48 HV, and the distal metaphysis hardness value was 35.24 ± 5.17 HV. The shaft was 23.5% harder than the proximal metaphysis and 20% harder than the distal metaphysis. The microhardness test demonstrated that the bone hardness value of the diaphysis was significantly higher than those of both the proximal and distal metaphysis of the radius (both *P* < 0.05). The mean pullout strength values ranged from 552 N (the distal metaphysis) to 2296 N (the diaphysis). The greatest pullout strength of the radius was observed for the shaft, with a pullout strength of 1727.96 ± 111.44 N. The pullout strength of the proximal metaphysis was 726.33 ± 236.39 N, and the pullout strength of the distal metaphysis was 590.67 ± 36.30 N. The pullout strength of the shaft was 138% greater than that of the proximal metaphysis and 190% greater than that of the distal metaphysis. The pullout strength was also higher in the diaphysis than at both ends of the radius (both *P* < 0.05). A positive correlation was found between bone hardness and pullout strength (*R* = 0.927, *P* < 0.001).

**Conclusions:**

Bone hardness and screw pullout strength are higher in the diaphysis of the radius than at either end. The pullout strength is positively related to bone hardness in the human radius.

## Introduction

Radial and ulnar fractures account for 6.3% of fractures of the body^1^. A study reported that in the United States, distal radial fractures accounted for more than 640 000 of cases during 2001 alone, representing one of the most common types of fractures^2^. Surgeons understand that the epidemiology of radial fractures can help them choose the most appropriate treatment options. The influence of environmental and lifestyle factors on the risk of radial fractures has recently been evaluated to further examine the reasons for the increasing rates and to reduce the dysfunction of radial fractures, which can usually be treated by internal fixation. Implant failure as a result of screw loosening is a serious complication after internal fixation of bone fractures^3^.

Many factors affect the ability to use internal fixation to treat radial fractures. Pullout strength is one of the most important issues in internal fixation stabilization, which can be used to measure the screw fixation strength^4^. The pullout strength is affected by both screw properties and bone properties. Screw properties include the screw design, the length of the screw, the insertional torque, and the pilot hole preparation. Bone properties consist of the properties of the bone material and the anatomic characteristics of the position where the screw is being inserted. The bone mineral density (BMD) is an important property that affects the biomechanics of screw fixation. Tingart *et al.* 5 studied the regional variability using computed tomography, which revealed a co‐direct relationship between the bone mineral density (BMD) and pullout strength. Osteoporotic bone and poor bone quality may lead to screw loosening and inhibit bone healing^6^. Other factors that affect pullout strength include the screw insertion technique. However, the correlation between the insertional torque and the pullout strength of the screw has not since been investigated in great detail.

Bone hardness is characterized by resistance to penetration and perpetual indentation^7^, and correlated with the degree of mineralization, which was calculated for both cortical and trabecular bone tissues for each indentation location. In general, there are two types of bone tissue: trabecular bone and cortical bone. Trabecular bone is a highly porous structure that fills the proximal and distal ends of all long bones. Cortical bone is the dense tissue that forms the outer shell of the long bones.

Bone hardness is one of the most important features of bone, which encompasses elastic deformation and plastic deformation. Macrohardness, microhardness, and nanohardness tests have been widely used to evaluate the properties of bone on these different scales. The macroscale mechanical properties of bone are controlled by both the structural organization of the microscale and nanoscale constituents as well as the intrinsic mechanical properties of these constituents across the different length scales^8^.

The Vickers hardness test is widely used and provides a convenient method for carrying out nondestructive measurements of the resistance of a material to plastic deformation^9^. It is believed that bone hardness measured by Vickers indentation is an important methodology for the evaluation of bone mechanical properties at the bone structural unit (BSU) level.

Some previous studies have focused on the hardness of bone. A study conducted by Hodgskinson *et al.* 10 showed that the cortical bone hardness value was 10%–20% higher than that of trabecular bone. Zysset *et al.* 11 reported similar results, in which the trabecular and cortical lamellae hardness values were measured by using nanoindentation technology. However, Weaver *et al.* 12 found that the hardness value of cortical bone was generally somewhat greater than that of trabecular bone, which clearly differed from the findings of the current study. Katoh *et al.* 13 reported the distribution of the patellar bone hardness value. In their study, the hardness in the lateral facet and the proximal and central regions was higher than that in the medial facet and distal regions. Nakabayashi *et al.* 14 investigated the hardness of the distal femur, and they demonstrated that bone hardness decreased sharply over the first two levels below the surface. Based on data obtained from one cadaver, Ohman *et al.* 15 found that cortical bone was harder than trabecular bone in the human radius.

Although many studies have examined pullout strength, few studies have focused on the relationship between bone hardness and pullout strength. In addition, little is known about the relationship between bone hardness and the pullout strength of the human radius. Therefore, this study had two aims: (i) to determine whether there are certain distribution rules for the hardness and pullout strength of the radius; and (ii) to determine whether there is a positive correlation between bone hardness and screw pullout strength.

## Materials and Methods

### *Sample Preparation*

This study was approved by the institutional review board of the Third Hospital of Hebei Medical University and registered at the WHO International Clinical Trials Registry Platform under number ChiCTR‐BPR‐17010818. Three fresh, unembalmed human cadavers were obtained from the anatomy department of Hebei Medical University: two male and one female, aged 45, 58, and 62 years, with heights of 172 cm, 170 cm, and 155 cm and weights of 80 kg, 76 kg, and 60 kg, respectively. The three donors had no systemic or local diseases that affected the bone.

All radiuses were examined by X‐ray to exclude bone pathology. Three radiuses were freshly harvested, the soft tissues were removed, and the bones were stored at −20°C to preserve the physical properties of the bone^16^. Three unilateral radius bones were obtained randomly from three donors and were used to perform the bone hardness test. The other three unilateral radiuses were used to perform the pullout testing. The radiuses were divided into proximal metaphysis, diaphysis, and distal metaphysis. The proximal metaphysis was divided into the head, neck, and radial tuberosity, and the distal epiphysis included the palmaris radius and styloid process. The shaft of the radius was divided equally into nine segments. Each radius was sawed by a band saw into 14 parts, which were prepared for the microindention testing. The microindention testing sample precision cuts were performed with a Buehler Isomet 11‐1280‐250 low‐speed diamond saw (Buehler, Ltd., USA). Each microindention sample was cut at a thickness of 3 mm and fixed on a glass sheet with epoxy resin. The sample surface was polished with progressive grades of sandpaper and finished with a 0.25 μm diamond slurry. Constant cooling liquid was used to cool the samples during the cutting operations. Once the procedure was completed, the samples were stored at −20°C until microindention testing.

### *Microindentation Testing*

**Figure 1:**
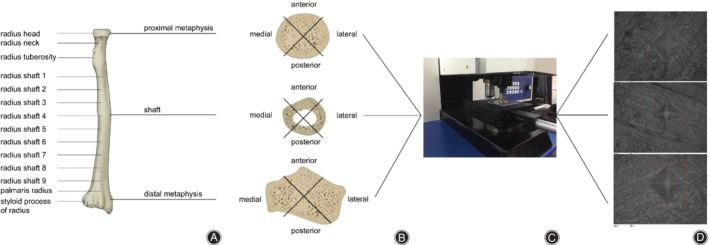
Microindentation testing of the human radius procedure. (A) The radiuses were divided into proximal metaphysis, diaphysis, and distal metaphysis, resulting in a total of 14 parts. (B) Microindention testing of the radius samples. (C) Microindention testing of the radiuses. (D) Microindention picture of the radiuses.

Microindentations on a bone sample surface under wet conditions were generated using a Vickers diamond indenter^17^. Microindentation was performed on each bone sample surface using a Vickers microhardness tester (Model KB5BVZ‐Video, Stuttgart, Germany), and the hardness was measured as the hardness value (HV, 1 HV = kgf/mm^2^). Before testing, all samples were immersed in Ringer's solution for 1 h to assure rehydration of the bone tissue^18^. Twenty indentions were randomly performed for each sample, which were equally divided into four quadrants (anterior, medial, posterior, and lateral). Hence, a total of 840 microindentations were performed on the three radiuses. Before indention, each sample was viewed under an optical microscope to ensure the bone surface was intact and not damaged. According to the standard test method from the American Society for Testing Material and previous studies^10^, the microindentations were performed on each sample with a load of 50 gf. The indentation time was set to 12 s (Fig. 1). The hardness value (HV/0.05) was computed for each indentation. The lengths of the diagonals were measured under reflected light microscopy, and the Vickers hardness value was calculated. According to a study by Ziv and colleagues^19^, indentations in which one diagonal was 10% or more longer than the other were ignored, and the indention was repeated. These preliminary data were used to determine the appropriate sample size of microindentations to be performed on each bone segment.

### *Pullout Strength Testing*

**Figure 2:**
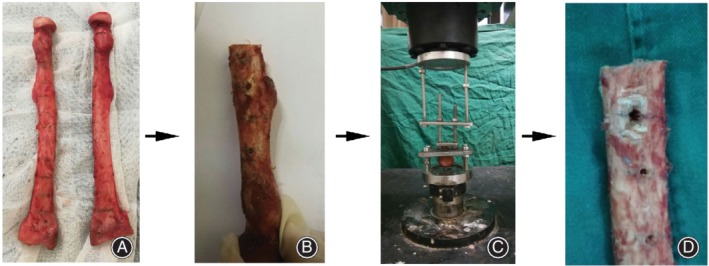
Pullout strength testing of the human radius procedure. (A) The radiuses were divided into the proximal metaphysis, the diaphysis, and the distal metaphysis, resulting in a total of 14 parts. (B) Pullout strength testing of the radius samples. (C) Pullout strength testing of the radiuses. (D) The radius samples after pullout strength testing.

The other three radiuses were collected from the other side of the three donors. The radiuses were marked with a surgical pen to divide them equally into 14 segments with a length of 2 cm. The proximal radius was divided into three segments, the shaft was divided into nine segments, and the distal radius was divided into two segments. Each bone segment was cut perpendicular to the longitudinal axis. The bone segments were drilled to create a pilot hole of 2.5 mm in diameter, and stainless steel screws (diameter, 3.5 mm; length, 42 mm) were inserted perpendicular to the surface^20^. Bone segments were fixed by using a device, and pullout strength tests were performed on an 8874 Universal Testing Machine (Shimadzu AG‐A20000, Shanghai, China). The pullout tests were performed with a preload of 50 N and speed of 10 mm/s. Data were collected every 0.005 s. The failure load and failure model were recorded for each pullout test. The pullout strength of each specimen was recorded (Fig. 2).

### *Statistical Analysis*

Data analysis was performed using SPSS19.0 (SPSS Inc., Chicago IL, USA). Data for bone hardness and pullout strength were checked for normality using the Kolmogorov–Smirnov test. For data with a normal Gaussian‐shaped distribution, data were shown as the mean ± standard deviation. The radius hardness and pullout strength distribution among three donors were analyzed by using an analysis of variance randomized block design. The correlation between the radius hardness value and pullout strength was tested by Pearson relevant analysis. For all statistical analyses, *P <* 0.05 was considered statistically significant.

## Results

### *The Distribution of Bone Hardness in the Human Radius*

**Figure 3:**
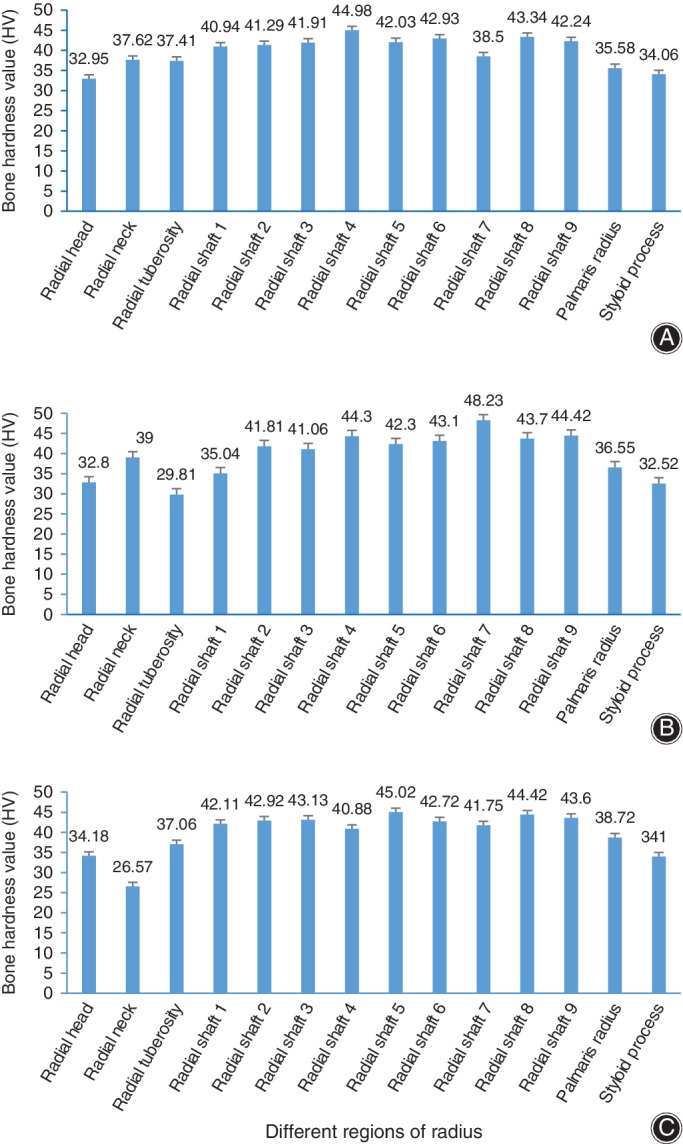
Bone hardness values in different regions of the radius: (A) Donor A; (B) Donor B and (C) Donor C.

The bone hardness distribution of the radius is shown in Table 1 and Fig. 3. The mean hardness value ranged from 33.30 HV (the head) to 43.82 HV (the diaphysis). The hardest part of the radius was the shaft, with a hardness value of 42.54 ± 5.59 HV. The proximal metaphysis hardness value was 34.15 ± 6.48 HV, and the distal metaphysis hardness value was 35.24 ± 5.17 HV. The shaft was 23.5% harder than the proximal metaphysis and 20% harder than the distal metaphysis.

**Table 1:** Bone hardness values in different regions of the radius (HV; $\bar{x}$ ± S)

	Proximal metaphysis			Diaphysis									Distal metaphysis		*P*_1_	*P*_2_	*P*_3_
Groups	Radial head	Radial neck	Radial tuberosity	Radial shaft 1	Radial shaft 2	Radial shaft 3	Radial shaft 4	Radial shaft 5	Radial shaft 6	Radial shaft 7	Radial shaft 8	Radial shaft 9	Palmaris radius	Styloid process			
Donor A	32.95 ± 3.82	37.62 ± 5.91	37.41 ± 2.97	40.94 ± 2.84	41.29 ± 5.03	41.91 ± 6.39	44.98 ± 5.92	42.03 ± 5.58	42.93 ± 5.48	38.50 ± 4.97	43.34 ± 4.56	42.24 ± 4.15	35.58 ± 4.74	34.06 ± 3.55			
Donor B	32.80 ± 3.12	39.00 ± 6.07	29.81 ± 8.73	35.04 ± 5.67	41.81 ± 4.68	41.06 ± 6.80	44.30 ± 6.49	42.30 ± 5.77	43.10 ± 5.61	48.23 ± 3.98	43.70 ± 5.30	44.42 ± 4.06	36.55 ± 5.84	32.52 ± 4.92			
Donor C	34.18 ± 3.85	26.57 ± 4.26	37.06 ± 6.35	42.11 ± 4.55	42.92 ± 4.84	43.13 ± 6.16	40.88 ± 3.84	45.02 ± 5.81	42.72 ± 5.99	41.75 ± 3.84	44.42 ± 5.87	43.60 ± 4.58	38.72 ± 2.95	34.00 ± 6.31			
Average of region	33.30 ± 3.60	34.40 ± 7.77	34.76 ± 7.27	39.36 ± 5.42	42.00 ± 4.82	42.03 ± 6.40	43.39 ± 5.73	43.11 ± 5.79	42.91 ± 5.60	42.82 ± 5.87	43.82 ± 5.20	43.42 ± 4.29	36.95 ± 4.77	33.52 ± 5.02			
Average of part	34.15 ± 6.48			42.54 ± 5.59									35.24 ± 5.17		0	0.11	0

*P*_1_: proximal metaphysis *vs* diaphysis; *P*_2_: proximal metaphysis *vs* distal metaphysis; *P*_3_: distal metaphysis *vs* diaphysis

The hardness was significantly enhanced in the diaphysis than the metaphysis in the radius (*P* < 0.001). However, no significant difference was found between the proximal (34.15 HV) and distal (35.24 HV) ends of the radius (*P* = 0.110).

### *The Distribution of Pullout Strength in the Human Radius*

**Figure 4:**
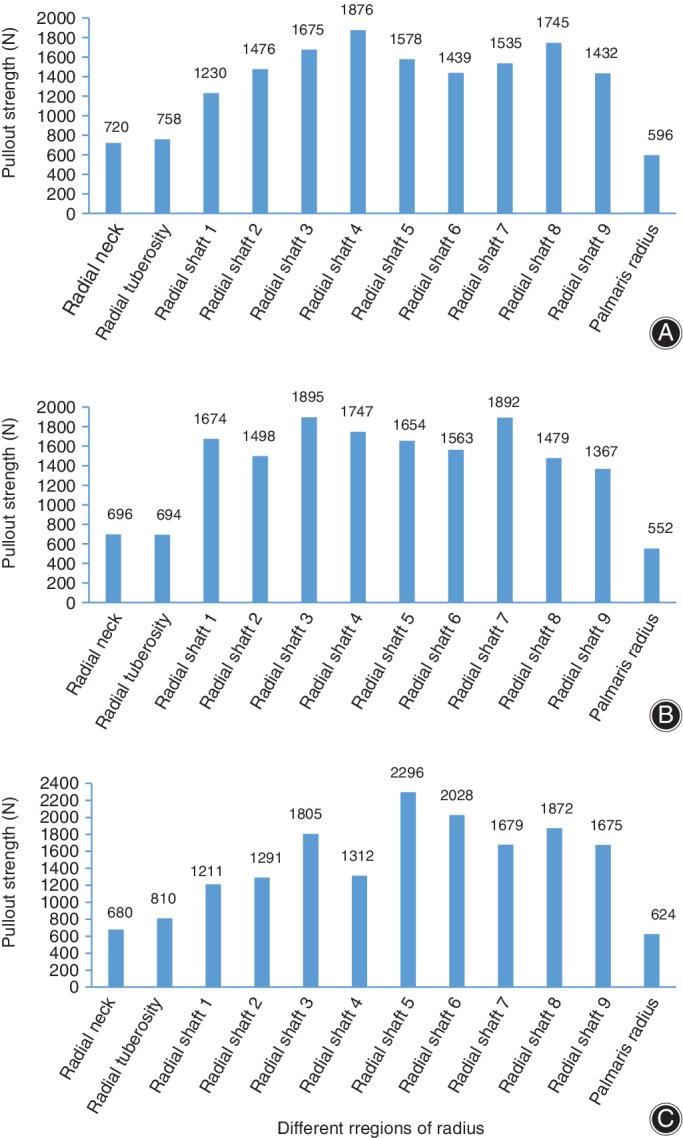
Screw pullout strength in different regions of the radius: (A) Donor A; (B) Donor B and (C) Donor C.

The pullout strength is summarized in Table 2 and Fig. 4. The mean pullout strength value ranged from 552 N (the distal metaphysis) to 2296 N (the diaphysis). The greatest pullout strength of the radius was in the shaft, with a pullout strength value of 1727.96 ± 111.44 N. The pullout strength of the proximal metaphysis was 726.33 ± 236.39 N, and the pullout strength of the distal metaphysis value was 590.67 ± 36.30 N. The pullout strength value of the shaft was 138% greater than that of the proximal metaphysis and 190% greater than that of the distal metaphysis.

**Table 2:** Screw pullout strength in different regions of the radius (N, $\bar{x}$ ± S)

	Proximal metaphysis		Diaphysis									Distal metaphysis	*P*_1_	*P*_2_	*P*_3_
Groups	Radial neck	Radial tuberosity	Radial shaft 1	Radial shaft 2	Radial shaft 3	Radial shaft 4	Radial shaft 5	Radial shaft 6	Radial shaft 7	Radial shaft 8	Radial shaft 9	Palmaris radius			
Donor A	720	758	1230	1476	1675	1876	1578	1439	1535	1745	1432	596			
Donor B	696	694	1674	1498	1895	1747	1654	1563	1892	1479	1367	552			
Donor C	680	810	1211	1291	1805	1312	2296	2028	1679	1872	1675	624			
Average of region	698.67 ± 20.13	754.00 ± 58.10	1371.67 ± 262.00	1421.67 ± 113.69	1791.67 ± 110.60	1645.00 ± 295.51	1842.67 ± 394.43	1676.67 ± 310.52	1702.00 ± 179.61	1698.67 ± 200.56	1491.33 ± 162.35	590.67 ± 36.30			
Average of part	726.33 ± 236.39		1727.96 ± 111.44									590.67 ± 36.30	0	0.406	0

*P*_1_: proximal metaphysis *vs* diaphysis; P_2_: proximal metaphysis *vs* distal metaphysis; *P*_3_: distal metaphysis *vs* diaphysis

The screw pullout strength in the shaft (1727.96 ± 111.44) was significantly enhanced compared with those of both ends (*P* = 0.001; *P* = 0.003). However, significant differences were not found between the proximal (726.33 ± 236.39) and distal metaphysis (590.67 ± 36.30) (*P* = 0.927). The pullout strength test for the head and styloid process of the radius failed, and no valid data were obtained.

### *The Relationship Between Bone Hardness and Pullout Strength in the Human Radius*

**Figure 5:**
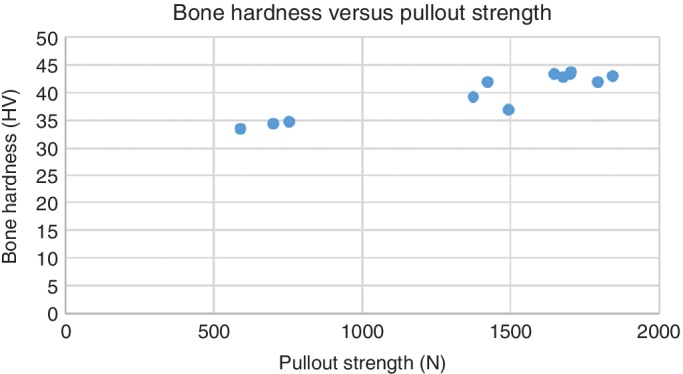
Bone hardness versus pullout strength of the human radius. Pullout strength is positively related to bone hardness in the human radius.

The statistical analysis showed a high linear correlation between the bone hardness and screw pullout strength in the human radius (*R* = 0.927, *P* < 0.001). The data illustrated in Fig. 5 show a scatter diagram with a best‐fit line.

## Discussion

The current study identified the distribution rules of bone hardness and pullout strength in the human radius. The hardness of the shaft of the radius was significantly higher than that at both ends. The data obtained from the hardness and pullout strength tests showed a strong correlation between bone hardness and pullout strength. The greatest pullout strength was determined in the center of the shaft and the lowest at the distal epiphysis.

The authors considered some reasons to explain the data obtained in this study. Bone hardness is characterized by resistance to penetration and permanent indentation. Bone hardness is determined by the composition and structure of all levels of the material, such as the nanostructural (lamella), microstructural (osteon/trabecular packet), and structural (compact/trabecular) organization. First, much of the difference is likely produced by variations in the mineral content. The composition (e.g. mineral and collagen) of bone may be responsible for the differences in hardness. Second, the Vickers microhardness indentions at microstructural, osteon or tabular levels were different. Bone consists of highly complex structures, which are described in terms of up to seven hierarchical levels of organization^21^. The seven hierarchical levels are major components (level 1), mineralized collagen fibrils (level 2), fibril arrays (level 3), fibril array patterns (level 4), cylindrical motifs (osteons, level 5), spongy versus compact bone (level 6), and whole bone (level 7). The Vickers microhardness indentions were performed at bone osteons or bone trabecula, which represent level 5. Therefore, the hardness identified in level 1 to 4 structures may be different. Third, different bone remodeling rates or greater bone mineral density have been found in cortical bone compared with trabecular bone^22^. Furthermore, the mineralization of cortical bones is significantly greater than that of trabecular bones^23^. Bone hardness is anisotropic, which implies a hardness difference when measured in different directions.

Based on the data obtained from the pullout strength tests, the diaphysis had a greater pullout strength than the epiphysis of the human radius. The difference between cortical and spongy bone could explain the results; however, due to the small sample size, we could not identify the distribution rule in the diaphysis or epiphysis. In a future study, the sample size will be increased to obtain more detailed rules.

The rules of distribution of bone hardness and pullout strength in the human radius were similar, and there was a positive correlation between them based on the relevant analysis in the present study. Reitman *et al.* 24 report a maximum pullout strength value of approximately 70% to 90% of the maximal torque of screw insertion. In this study, the bone mineral density and cortical thickness were not significantly different between samples. However, in our study, we cannot exclude the effects of cortical thickness and bone mineral density difference on the pullout strength, which might be the major cause of our inability to determine the pullout strength rules in cortical or cancellous bone.

In the present study, although we identified the effects of bone hardness on the pullout strength of the human radius, some limitations should be mentioned. First, we did not take other factors affecting pullout strength, such as bone cortical thickness, BMD, and screw characteristics, into consideration, although Reitman *et al.* 24 reported that the pullout strength was not significantly correlated with cortical thickness. Second, the sample size was small, which might negatively impact the conclusions; however, the tendency of the data were obvious. In future, studies with a large sample size should be performed to confirm the conclusions drawn in this study. The illustration of the distribution of bone hardness in the radiuses helps us to understand internal fixation mechanics and provides a basis for the rational design of prostheses and internal fixations. The data for pullout strength may help to improve surgical techniques and prevent implant loosening of bone fractures.

### *Conclusion*

Bone hardness and screw pullout strength were higher in the diaphysis of the radius than at both ends. Pullout strength is positively related to bone hardness in the human radius. Further investigation is needed to clarify the mechanism responsible for differences in bone hardness and pullout strength.
